# Assessment of Preparation Methods for Organic Phosphorus Analysis in Phosphorus-Polluted Fe/Al-Rich Haihe River Sediments Using Solution ^31^P-NMR

**DOI:** 10.1371/journal.pone.0076525

**Published:** 2013-10-15

**Authors:** Wenqiang Zhang, Baoqing Shan, Hong Zhang, Wenzhong Tang

**Affiliations:** 1 State Key Laboratory on Environmental Aquatic Chemistry, Research Center for Eco-Environmental Science, Chinese Academy of Science, Beijing, P.R. China; 2 University of Chinese Academy of Science, Beijing, P.R. China; Plymouth University, United Kingdom

## Abstract

Fe/Al-rich river sediments that were highly polluted with phosphorus (P) were used in tests to determine the optimum preparation techniques for measuring organic P (Po) using solution ^31^P nuclear magnetic resonance spectroscopy (^31^P-NMR). The optimum pre-treatment, extraction time, sediment to solution ratio and sodium hydroxide-ethylenediaminetetraacetic acid (NaOH-EDTA) extractant solution composition were determined. The total P and Po recovery rates were higher from freeze- and air-dried samples than from fresh samples. An extraction time of 16 h was adequate for extracting Po, and a shorter or longer extraction time led to lower recoveries of total P and Po, or led to the degradation of Po. An ideal P recovery rate and good-quality NMR spectra were obtained at a sediment:solution ratio of 1∶10, showing that this ratio is ideal for extracting Po. An extractant solution of 0.25 M NaOH and 50 mM EDTA was found to be more appropriate than either NaOH on its own, or a more concentrated NaOH-EDTA mixture for ^31^P-NMR analysis, as this combination minimized interference from paramagnetic ions and was appropriate for the detected range of Po concentrations. The most appropriate preparation method for Po analysis, therefore, was to extract the freeze-dried and ground sediment sample with a 0.25 M NaOH and 50 mM EDTA solution at a sediment:solution ratio of 1∶10, for 16 h, by shaking. As lyophilization of the NaOH-EDTA extracts proved to be an optimal pre-concentration method for Po analysis in the river sediment, the extract was lyophilized as soon as possible, and analyzed by ^31^P-NMR.

## Introduction

Organic P (Po) is an important phosphorus fraction in river sediments, and it can be directly used or mineralized into inorganic P (Pi) by aquatic organisms [1]. Despite the importance of Po in sediments, Po fractions and their transformation mechanisms remain poorly understood, mainly because of analytical limitations and complexity of the Po constituents that are present [2], [3]. Solution ^31^P nuclear magnetic resonance spectroscopy (^31^P-NMR) is an ideal tool for analyzing Pi and Po, as was first shown by Newman and Tate [2], [4] when they analyzed soil extracts.

Po must be extracted from the soil or sediment sample before it can be qualitatively or quantitatively analyzed. The extraction process must, of course, remove as much Po from the soil or sediment as possible, and it must also allow the Po components to remain untransformed. The extractant and the extraction conditions used are, therefore, key factors in determining the effectiveness of the analysis. Many chemicals have been used as Po extractants, including NaOH [4], Bu_4_NOH [5], Chelex [6], NaOH-Chelex [7], NaOH and NaF [8], NaHCO_3_ [9], NaHCO_3_+Na_2_S_2_O_4_ (BD) [10], and H_2_SO_4_ [9]. Cade-Menun and Preston [11] examined various extractants and concluded that a mixture of NaOH and ethylenediaminetetraacetic acid (EDTA) was an ideal extractant that gave excellent and stable recovery of the various P components. The presence of the chelating agent, EDTA, along with an organometallic component increased the extraction efficiency of the NaOH [11]. Based on this conclusion, a one-step extraction procedure using a mixture of NaOH and EDTA was considered to be particularly suitable for the analysis of Po in most soils [3]. Several P-containing compounds have been detected using this extractant mixture, including phosphonate (phon-P), orthophosphate monoesters (mono-P; comprising inositol phosphates, phosphoproteins and mononucleotides), orthophosphate diesters (diester-P; comprising phospholipids and DNA; lipid-P and DNA-P), pyrophosphate (pyro-P), and polyphosphate (poly-P) [9], [12]–[14].

Although the NaOH-EDTA extractant and extraction procedure have been widely used, the NaOH and EDTA proportions and the extraction procedures have often been chosen arbitrarily [15]. For example, combinations of 0.25 M NaOH with 50 mM EDTA [16] and 0.5 M NaOH with 0.1 M EDTA [17] have been used, and one-step extractions have used sediment:solution ratios of 1∶3 (V:V) [18], 1∶6 (W:V) [19], 1∶10 (W:V) [20], 1∶20 (W:V) [21], 1∶30 (W:V) [22] and 1∶50 (W:V) [23]. The extraction time, including shaking, has usually been 16 h [24], but a shorter extraction time (6 h) has also been reported [11]. Extracts have often been concentrated by freeze drying [16], [18], [20], [25], [26]. The extractant and extraction procedure used are critical to the P recovery and the reliable determination of individual P components, but the physical and chemical properties of soils and sediments vary widely, so the analytical method has to be adjusted to suit a particular set of samples.

The Haihe River Basin is affected by a complex mixture of pollutants, and the P concentrations are high in most of the rivers entering the basin because of point and diffuse pollution sources, such as wastewater inputs. The aim of this study was to establish the optimum extractant mixture and extraction procedure for analyzing Po in river sediment samples. To accomplish this, we analyzed sediment samples from the highly P-polluted Haihe River Basin, and determined the optimum sample preparation method parameters, including the composition of the NaOH-EDTA extractant, extraction time, sediment:solution ratio and the pre-treatment technique.

## Materials and Methods

### Sediment Sampling

The Haihe River Basin, in northern China, has an area of about 300,000 km^2^ and a population of 145 million. The river basin is more than 45% urbanized, and it contains Beijing, Tianjin, and many other cities. The Fuyang River (36°23′–38°14′ N, 114°19′–116°7′ E) is an important contributor to the Haihe River system, and its main channel is 402 km long, passing through the cities of Hengshui, Handan, and Xingtai. The Wangyang Ditch, the Shaocun Ditch and the Xiao River are important tributaries of the Fuyang River, and they receive large amounts of domestic wastewater from Shijiazhuang, the capital of Hebei Province. The Niuwei River is another important tributary of the Fuyang River system, and is mainly within the Xingtai area. The upstream part of the Fuyang River is in the Handan area, which is famous for its steel industry. In 2007, a total of 0.52 billion t of wastewater, comprising equal amounts of domestic sewage and industrial wastewater, were discharged into the river. The high level of pollutant discharges into the Fuyang River has led to poor water quality, and P is the primary pollutant in most of the Fuyang River system. The Fuyang River was selected for this study because of the extreme P pollution and because it is representative of many rivers in northern China.

Sediment samples were collected from five sampling sites in the Fuyang River system as follows: site 1, 36°44′27.60″ N, 114°52′55.20″ E; site 2: 37°30′3.60″ N, 115°3′7.20″ E; site 3: 37°30′50.40″ N, 115°4′19.20″ E; site 4: 37°33′14.40″ N, 115°9′14.40″ E; and site 5: 37°41′31.20″ N, 115°37′37.20″ E. All of the locations where the samples were taken were publically owned, and no permits were required for the field studies described. There were no endangered or protected species in the study area. Three surface (about 5 cm deep) sediment samples, from locations not less than 500 m apart, were collected from each site using a Peterson grab sampler. Samples were pooled and homogenized to give a representative sample. Part of each sample was immediately frozen and stored at −18°C in the field, and the remaining fresh sample was sealed in a plastic bag for analysis as soon as was possible. The frozen sediment samples were freeze-dried on return to the laboratory, then homogenized and impurities removed. Each sample was then ground and a representative sample for analysis was obtained using the quartering method.

### Analysis of Sediment Properties

The sample pH was determined in a sediment suspension in deionized water (at a sediment:water ratio of 1∶2.5). The organic matter (OM) content of each sediment sample was determined by loss on ignition at 550°C for 4 h [27], and the total Al, Ca, Fe, Mg and Mn contents were measured by inductively coupled plasma-optical emission spectroscopy (ICP-OES, PerkinElmer: Optima 8300, USA) after digestion in a HNO_3_-HCl-HF mixture (using a MARSXpress digestion system; CEM, Matthews, NC, USA). The total P (TP) content was determined by 1 mol L^−1^ HCl extraction (16 h) after pretreatment for 2 h at 500°C. Pi was determined by extracting the sediment with 1 M HCl for 16 h. The Po concentrations were calculated as the difference between TP and Pi [28]. TP in surface water samples was measured using the molybdenum blue method [29].

### Optimization of the Extraction Procedure

#### Sediment preparation

Freeze-dried, air-dried and fresh samples were extracted with 0.25 M NaOH and 50 mM EDTA, using a sediment:solution ratio of 1∶10 (W:V). The extraction procedure involved shaking the samples for 16 h at 25°C, then centrifuging at 9462×g (RCF)(Beckman Coulter: Avanti J-26XP, USA) before removing the supernatants for ^31^P-NMR analysis.

#### NaOH-EDTA extractant composition

Freeze-dried sediment samples were extracted using solutions containing different proportions of NaOH and EDTA, at a sample:solution ratio of 1∶10 (W:V), with an extraction time of 16 h, at room temperature. The NaOH concentrations tested were 0.1, 0.25, 0.5 and 1.0 M, and the EDTA concentrations tested were 0, 25, 50, 75 and 100 mM. The following 4 extracts were chosen for the ^31^P-NMR analysis tests: 0.25 M NaOH, 0.5 M NaOH with 100 mM EDTA, 0.25 M NaOH with 50 mM EDTA and 1 M NaOH with 50 mM EDTA.

#### Extraction ratio

Freeze-dried sediment samples were extracted with 0.25 M NaOH and 50 mM EDTA by shaking for 16 h at room temperature, with sediment:solution ratios of 1∶5, 1∶8, 1∶10, 1∶15, 1∶20 and 1∶30 (W:V). The extracts, with sediment:solution ratios of 1∶5, 1∶10, 1∶20 and 1∶30 were then freeze-dried for ^31^P-NMR analysis.

#### Extraction time

Freeze-dried sediment samples were extracted by shaking with 0.25 M NaOH and 50 mM EDTA at a sediment:solution ratio of 1∶10 (W:V), at 25°C and extraction times of 2, 4, 8, 12, 16, 24 and 32 h. The 2, 8, 16 and 24 h extracts were then freeze-dried for ^31^P-NMR analysis.

#### Pre-concentration technique

Freeze-dried sediment samples were extracted with 0.25 M NaOH and 50 mM EDTA, by shaking for 16 h at room temperature, with a sediment:solution ratio of 1∶10 (W:V). The extracts were freeze-dried for ^31^P-NMR analysis. Part of each freeze-dried extract was re-dissolved in 0.25 M NaOH for TP and Po analysis, and the re-dissolved extract was freeze-dried again for ^31^P-NMR analysis.

### ^31^P-NMR Analysis

An aliquot of each NaOH-EDTA extract was analyzed for Pi and TP using the molybdenum blue method, before and after digesting the 100-fold diluted sample (to avoid interference caused by EDTA) with potassium persulfate (K_2_S_2_O_8_) [30], [31]. The Po concentration in each extract was calculated as the difference between the Pi and TP concentrations. The remaining extract was frozen, lyophilized and used for ^31^P-NMR analysis. It has been shown that freezing an extract does not alter the P composition [14], [32].

Each lyophilized extract (300 mg) was re-dissolved in 0.6 mL D_2_O and 0.1 mL of 10 M NaOH, ultrasonicated for 30 min and then equilibrated for 5 min. A 2% (V:V) bicarbonate buffered dithionite solution (0.11 M NaHCO_3_ and 0.11 M Na_2_S_2_O_4_) was then added to the extract, to decrease interference in the ^31^P analysis from paramagnetic ions (Fe^3+^ and Mn^2+^). The supernatant was centrifuged for 15 min at 16873×g (RCF) (Eppendorf: Centrifuge 5418, Germany) and transferred to a 5 mm NMR tube.

Solution ^31^P-NMR spectra were obtained using a Bruker 400 MHz spectrometer (Bruker, Billerica, MA, USA) operating at 129.53 MHz at 25°C. We used a 90°C observation pulse, a relaxation delay of 2 s, and an acquisition time of 0.6 s. The spectra collected each contained approximately 20,000 scans, collected over a period of 14 h (at the Beijing Nuclear Magnetic Resonance Center). The chemical shifts were recorded relative to an 85% H_3_PO_4_ standard (δ = 0 ppm). Signals were assigned to P species based on data from the literature [2], [30]. Peak areas were calculated by visual inspection and using an automated peak analysis tool. The different P-species areas were used to calculate the contribution of each P compound group (diester-P (DNA-P and lipid-P), mono-P, ortho-P, phon-P, and pyro-P relative to the TP content in the NaOH-EDTA extract that had been determined using the molybdenum blue method. We verified the chemical shifts of the P compounds by carrying out spike experiments using known compounds to assign peaks (e.g., using Na_4_P_2_O_7_·10 H_2_O).

## Results and Discussion

### Sediment Properties

The properties of the sediment sample analyzed in this study are shown in Table 1. The TP concentration in the sediment was 6145.86±98.11 mg kg^−1^ and the Po concentration was 1598.35±23.09 mg kg^−1^, accounting for 26.01% of the TP concentration. The OM content of the sediment was 12.19±1.38%. There are many towns and farms along the Fuyang River, and large amounts of domestic, industrial and agricultural wastewater are released into the river. The sediment, therefore, accumulates a great deal of P, which is a common phenomenon in the Haihe River basin [32], [33], [34]. The pH of the sediment was 7.25±0.33, indicating that the sediment was slightly alkaline. The sediment had an unusual characteristic, in that the Fe and Al concentrations (55.59±1.23 and 29.12±2.79 g kg^−1^, respectively) were higher than the Ca concentration (16.17±2.87 g kg^−1^). The Mn and Mg concentrations in the sediment were 0.71±0.11 and 4.10±0.66 g kg^−1^, respectively.

**Table 1:** Properties of the sediment used in this study.

Parameter	Value
pH	7.25±0.33
Organic matter (%)	12.19±1.38
Total P (mg kg^−1^)	6145.86±98.11
Total organic P (mg kg^−1^)	1598.35±23.09
Fe (g kg^−1^)	55.59±1.23
Mn (g kg^−1^)	0.71±0.11
Al (g kg^−1^)	29.12±2.79
Ca (g kg^−1^)	16.17±2.87
Mg (g kg^−1^)	4.10±0.66

### The Influence of the Sediment Preparation Technique

**Figure 1:**
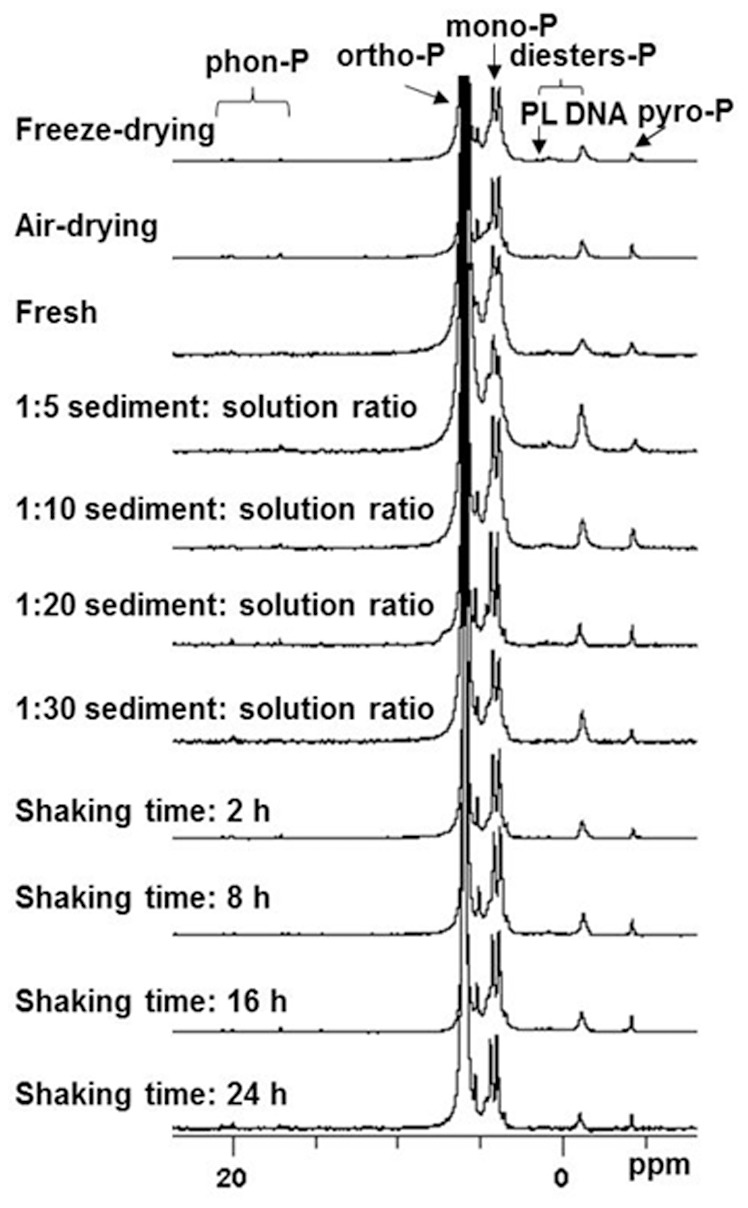
^31^P-NMR spectra of the sediment extracts after different analytical treatments.

The TP, Po and metal concentrations in the NaOH-EDTA extract differed depending on the sample pre-treatment (Table 2). The TP and Po concentrations were significantly higher in the freeze- and air-dried samples than in the fresh samples. The TP concentrations were 3711.28 mg kg^−1^ in the freeze-dried samples and 3687.24 mg kg^−1^ in the air-dried samples, while the Po concentrations were 879.29 mg kg^−1^ in the freeze-dried samples and 574.98 mg kg^−1^ in the air-dried samples. The TP concentrations were about 33 and 32% lower in the fresh samples than in the freeze- and air-dried samples, respectively, while the Po concentrations were about 42 and 11% lower in the fresh samples than in the freeze- and air-dried samples, respectively. These results were comparable to the results of other studies that used Fe/Al-rich sediments from lakes [15], [35]. The metal element concentrations also varied with the different pre-treatment methods. Ca, Fe and Mg extraction efficiencies were higher after freeze drying than with the other pre-treatment methods, but the Al concentration was highest in the fresh sample. The ^31^P-NMR analysis showed that the individual P groups were extracted more efficiently after freeze- and air-drying than from the fresh sediment samples. The ortho-P concentration in the freeze-dried sample extract (2797.8 mg kg^−1^) was higher than in the air-dried sample extract (3128.3 mg kg^−1^). The freeze-drying pre-treatment also resulted in the highest concentrations of DNA-P, lipid-P, mono-P, and pyro-P, at 65.9, 16.5, 796.6, and 25.1 mg kg^−1^, respectively (Table 3 and Fig. 1). Both the Pi and the Po recoveries were lower from the fresh sediment samples than from the freeze- and air-dried samples. Drying the sample will change the permeability and solubility of biological cells, and will increase the P extraction rate from the sediment. However, there is the risk that Po will be hydrolyzed during the drying procedure. Wang et al. showed that fresh samples were not suitable for Po analysis because of the heterogeneity of the sediment [36]. The Po concentration will also be underestimated when a sample is air-dried under warm conditions [37]. Our study showed that both the TP and the Po concentrations were lower when the samples were air-dried or fresh than when the samples were freeze-dried, so we concluded that freeze drying was an appropriate pre-treatment for river sediments.

**Table 2:** Concentrations† ‡ of total P (TP), organic P (Po), and metals in the NaOH-EDTA extract after different pre-treatments.

Pre-treatment	TP*	Po	Fe	Al	Ca	Mg		Mn
	mg kg^−1^		g kg^−1^					
Freeze drying	3711.28±98.2a**	879.29±16.2a	0.71±0.11a	0.15±0.01a	15.99±1.9a		0.29±0.01a	0.18±0.01a
Air drying	3687.24±132.7a	574.98±37.8a	0.32±0.09b	0.16±0.02a	14.59±2.2a		0.25±0.02b	0.14±0.01b
Fresh	2496.97±115.9b	512.24±29.9b	0.47±0.03c	0.36±0.01c	11.65±3.1b		0.22±0.01c	0.20±0.01c

† The concentration is based on the dry weight.

‡ The value was calculated from the concentration in the NaOH-EDTA extract and the dry weight.

* The values are the means and standard deviations of three replicate extracts.

** Different letters indicate significant differences using Tukey–Kramer’s mean comparison.

**Table 3:** Influence of pre-treatment on the concentrations^a^ (mg kg^−1^) of individual P components, analyzed by ^31^P-NMR.

Pre-treatment	Inorganic P		Organic P			
	Ortho-P	Pyro-P	Phon-P	Mono-P	Lipid-P	DNA-P
Freezedrying	2797.8 (75.4)^b^	25.1 (0.7)	9.4 (0.3)	796.6 (21.5)	16.5 (0.4)	65.9 (1.8)
Airdrying	3128.3 (84.8)	21.9 (0.6)	10.1 (0.3)	451.4 (12.2)	14.7 (0.4)	60.8 (1.6)
Fresh	1972.8 (79.0)	16.3 (0.7)	5.8 (0.2)	460.1 (18.4)	8.3 (0.3)	33.8 (1.4)

a The concentration is based on the dry weight.

b The figures in brackets are the proportions the individual P components provided to the total P concentrations in the NaOH-EDTA extracts.

DNA-P = deoxyribonucleic acids (orthophosphate diesters), Lipid-P = phospholipid (orthophosphate diesters), Mono-P = orthophosphate monoesters, Ortho-P = orthophosphate, Phon-P = phosphonate, Pyro-P = pyrophosphate.

### The Influence of the NaOH-EDTA Extractant Solution Composition

**Figure 2:**
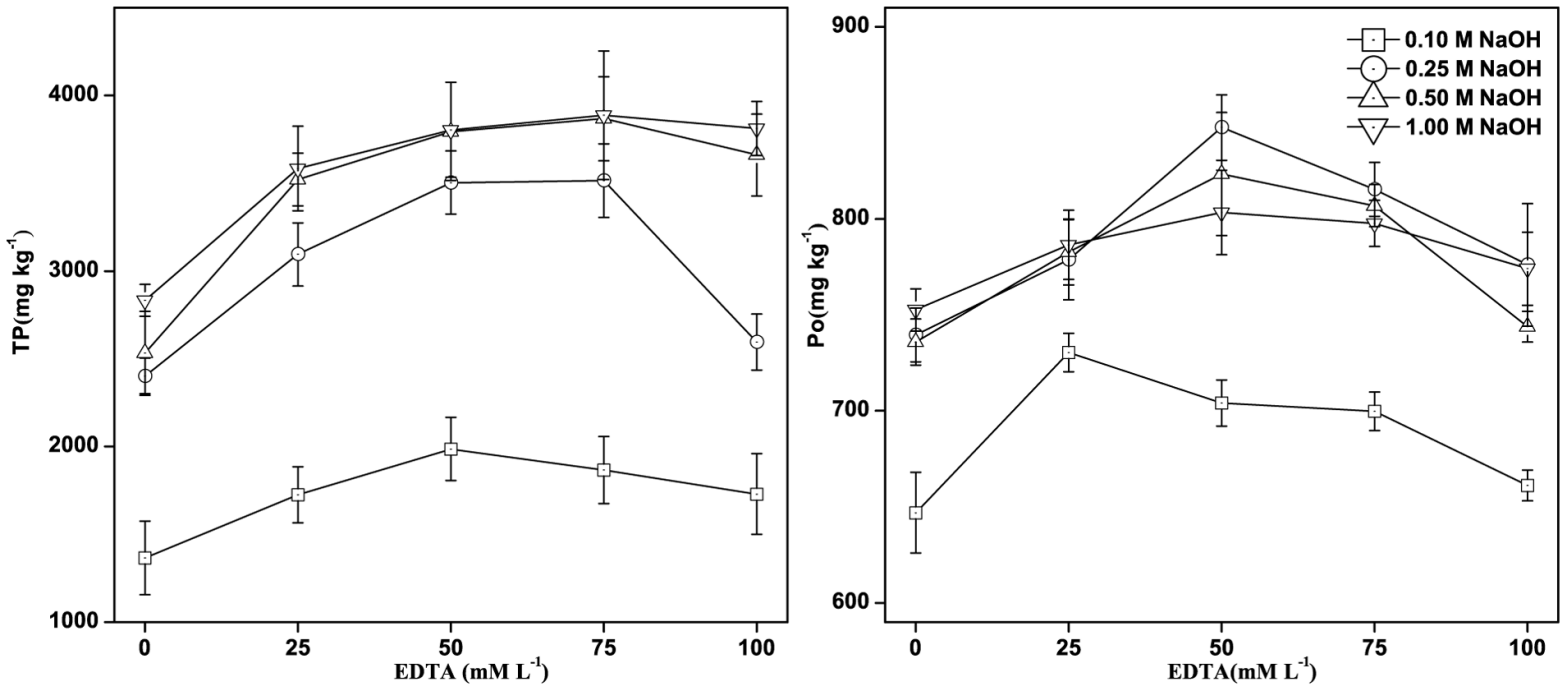
The total P (TP) and organic P (Po) concentrations found using different extractant solutions.

**Figure 3:**
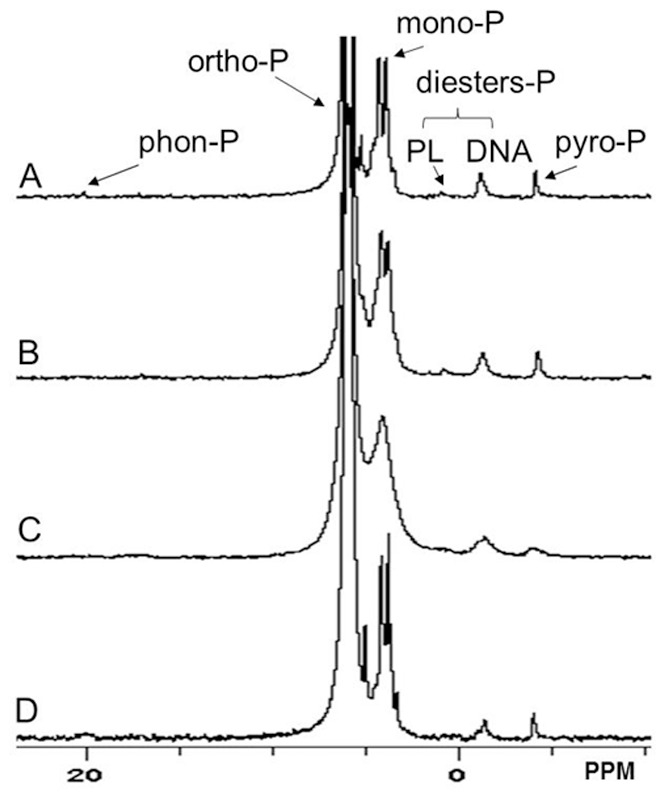
^31^P-NMR spectra for different extracts of the river sediments (A: 0.5 M NaOH and 100 mM EDTA; B: 0.25 M NaOH and 50 mM EDTA; C: 0.25 M NaOH; D: 1 M NaOH and 50 mM EDTA).

The composition of the NaOH-EDTA extractant solution has been shown to influence the extraction rate of P from soils and animal manures, but there has been little research on its effect on P extraction from river sediments [11], [38]. We found that the TP and Po concentrations varied with different NaOH-EDTA extractant compositions (Fig. 2). The TP and Po concentrations first increased and then decreased, with increasing EDTA concentration. A high NaOH concentration would, therefore, give better TP and Po recoveries than would a low NaOH concentration at the same EDTA concentration. The combinations of (1) 1.0 M NaOH with 75 mM EDTA solution and (2) 0.25 M NaOH with 50 mM EDTA solution gave good TP and Po recoveries, and resulted in TP and Po concentrations for these two extractant mixtures of 3886.45 and 847.64 mg kg^−1^, respectively. There was a strong positive correlation between pH and both the TP concentration (r^2^ = 0.56, *p*<0.01) and the Po concentration (r^2^ = 0.37, *p*<0.01) in the extracts, indicating that a high pH would lead to more P being released from the sediment. When only NaOH was used as the extractant, the TP and Po concentrations were lower than the concentrations when both NaOH and EDTA were used. The ^31^P-NMR spectra showed that there were six P species in the NaOH-EDTA extract, including DNA-P, lipid-P, mono-P, ortho-P, phon-P, and pyro-P (Fig. 3). The spectra for the NaOH-EDTA extracts were of better quality than those for the NaOH extracts. Lipid-P and phon-P were not detected in the NaOH extract. EDTA appeared to be an ideal complexing agent in the extracts, and chelated with the paramagnetic ions (such as Fe^3+^ and Mn^2+^) thereby improving the spectral quality. There were more free paramagnetic ions in the NaOH extract than in the NaOH-EDTA extract, which caused the NaOH extract spectra to be noisier than the NaOH-EDTA extract spectra. We found that the 0.25 M NaOH and 50 mM EDTA extractant was the most appropriate for analyzing Po in the sediment sample.

### The Influence of the Shaking Time

**Figure 4:**
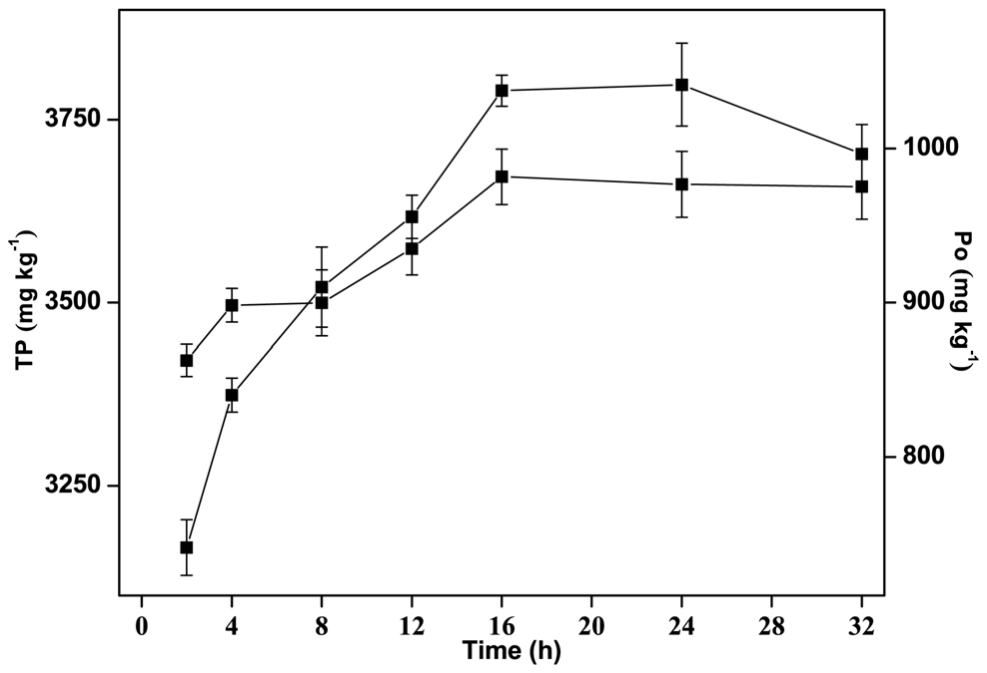
The total P (TP) and organic P (Po) concentrations found after different extraction times.

The extraction time needs to be sufficient for the sediment sample to remain mixed with the extractant so that most of the Po is extracted, but not long enough to allow the Po to become hydrolyzed. An appropriate extraction time is, therefore, vital to the Po analysis method. We extracted the freeze-dried sediment samples with 0.25 M NaOH and 50 mM EDTA at a sediment:solution ratio of 1∶10 (W:V) for different extraction times. The TP and Po extraction rates increased from an 8 h extraction time (Fig. 4), to their maximum at 16 h extraction. The TP extraction rate remained stable until a 32 h extraction time, indicating that a 16 h extraction time was sufficient for the EDTA to chelate with the Fe and Al and to allow the maximum possible amount of Fe/Al-bound P to be released from the sediment. The Po concentration in the extracts decreased slightly between 16 and 24 h, and then decreased markedly after an extraction time of between 24 and 32 h, indicating that some of the Po compounds were hydrolyzed after 16 h. The ^31^P-NMR spectra showed that the extraction rates for DNA-P, lipid-P, mono-P, and pyro-P increased between 2 and 16 h extraction time, but that the extraction rates for labile Po species, such as DNA-P and lipid-P, decreased after 16 h (Fig. 1). There is, therefore, the risk that Po may be hydrolyzed if the extraction time is too long. We found that an extraction time of 16 h was ideal for the Fe/Al-rich river sediments analyzed in this study (Fig. 4).

### The Influence of the Sediment: Solution Ratio

**Figure 5:**
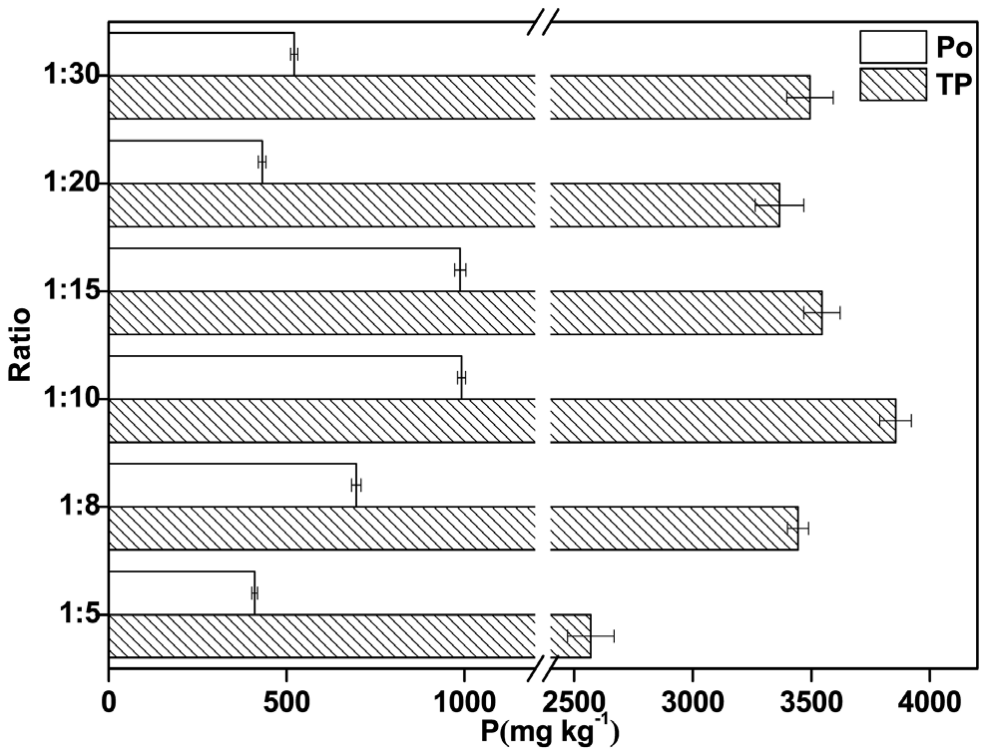
The total P (TP) and organic P (Po) concentrations found using different sediment:extractant solution ratios.

The influence of the sediment:solution ratio on the TP and Po concentrations is shown in Figs. 1 and 5. As the sediment:solution ratio increased, the TP and Po concentrations in the extracts first increased and then decreased. The TP and Po concentrations in the NaOH-EDTA extract increased linearly as the sediment:solution ratio was increased from 1∶5 to 1∶10, and the relationship between the TP and Po concentration with the sediment:solution ratio was described by the equations *y = *259.00 *x* – 1293.70 (r^2^ = 0.99, *p*<0.05) for TP and *y = *115.48*x* +176.05 (r^2^ = 0.99, *p*<0.05) for Po, where *y* is the analyte concentration and *x* is the sediment:solution ratio. These results showed that much more Fe/Al-bound P and some Po were extracted when more NaOH and EDTA were present, and this was consistent with the sediment P fractionation results, which showed that Fe/Al-bound P was the dominant P fraction in the river sediment we analyzed (unpublished data) [39]. It has been suggested that the TP:Po ratio will vary as pH varies, because of the different NaOH concentrations at different pH values [40]–[41]. The TP and Po extraction rates decreased as the sediment:solution ratio was increased from 1∶10 to 1∶30. The decrease in the Po extraction rate at higher sediment:solution ratios may have been due to the increase in the amount of NaOH present, leading to hydrolysis of some of the Po compounds.

### The Influence of Pre-concentration Techniques

**Figure 6:**
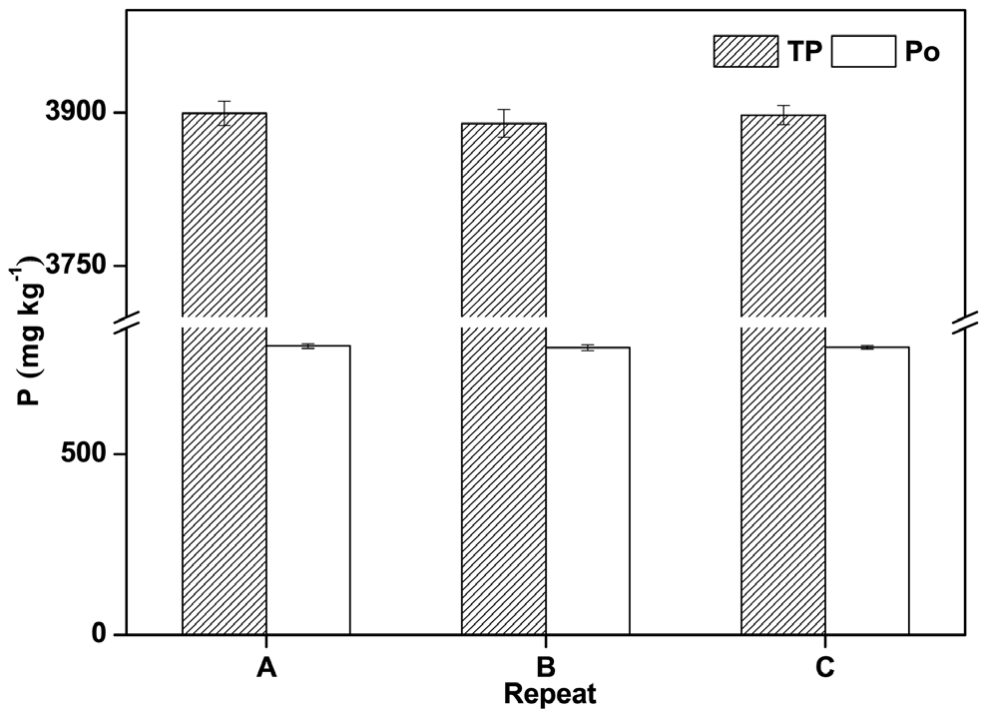
The total P (TP) and organic P (Po) concentrations in the re-dissolved extracts after lyophilization.

Pre-concentration techniques, including freeze-drying and rotary evaporation, were assessed. Po may decompose during pre-concentration, and there are few appropriate pre-concentration methods. As rotary evaporation has been studied in other studies [15], [42], we evaluated freeze-drying in this study. Triplicate NaOH-EDTA extracts were freeze-dried, and then the samples were re-dissolved with 0.25 M NaOH. TP concentrations in the triplicate re-dissolved samples were 3899.23±11.6, 3889.45±13.6, and 3897.66±9.3 mg kg^−1^, while Po concentrations were 799.84±6.5, 795.45±7.8, and 796.44±4.8 mg kg^−1^ (Fig. 6). The TP and Po concentrations in the original NaOH-EDTA extracts were 3923.78 and 797.46 mg kg^−1^, respectively, so less than 5.0% of either TP or Po was lost during freeze-drying and re-dissolution. The ^31^P-NMR spectra also showed that the Po composition was almost unchanged by the freeze-drying process. Freeze-drying was, therefore, found to be an appropriate pre-concentration method for analyzing Po in river sediments.

### General Optimization

The solution ^31^P-NMR was first used to analyze Po in soils, since which time it has been used to analyze marine and freshwater sediments [43], [44]. Although standard methods are difficult to establish for samples with complex physical-chemical properties, general extraction procedures and extractants (NaOH-EDTA) have been accepted [18], [35]. The Po concentrations in sediments are generally low, and there are often high concentrations of paramagnetic ions (such as Fe^3+^ and Mn^2+^) present. An ideal Po extraction procedure and extractant, therefore, would give a high Po recovery rate, low paramagnetic ion recovery rates, and would result in minimum alteration of the Po fractionation. The first stage of any analysis should therefore be optimization of the Po extractant to ensure it is suitable for the samples.

The extraction procedure parameters were chosen using literature sources, then modified to suit the characteristics of the river sediment being studied. We chose river sediment, rich in Fe and Al and with a high OM content, from a typical river that was highly polluted with P from wastewater inputs (unpublished data), like some other rivers in the Haihe River basin. Better analysis results were obtained for the freeze- and air-dried river sediment samples than for fresh samples. These results were comparable to results found for Lake Qinghai sediments [15]. However, Turner found that the differences between unreactive P (Po and pyro-P) concentrations extracted from fresh and air-dried samples were relatively small [38]. In the present study, the freeze-drying pre-treatment not only maintained the original Po fractionation and gave high TP and Po recoveries, but was also convenient for batch handling samples. Freeze drying is, therefore, the ideal choice for analyzing Po in river sediments from northern China.

The extraction time, sediment:solution ratio and pre-concentration method were critical parameters in the Po extraction method. An extraction time (with shaking) of 16 h was adequate for extracting TP and Po from the Fe/Al-rich sediment samples in this study, and shortening or extending the extraction time would have decreased the TP and Po recoveries, as has also been observed when analyzing Po concentrations in soils and wetland sediments [20], [45]. Sediment:solution ratios of between 1∶3 and 1∶50 have been used [18], [23], and, in our study, appropriate TP and Po recoveries were found with a sediment:solution ratio of 1∶10. A sediment:solution ratio of 1∶10 has also been used to extract Po from artificial lake sediments [20]. Rotary evaporation and lyophilization have been used to pre-concentrate NaOH-EDTA extracts [20], [42]. We tested lyophilization for pre-concentrating our NaOH-EDTA extracts, and found that the TP and Po concentrations and the Po component compositions were not altered during the freeze-drying process. Using 0.25 M NaOH and 50 mM EDTA as the extractant, high Po recoveries were achieved, and the Fe and Mn concentrations were optimum for the final ^31^P-NMR analysis. Using 1 M NaOH and 75 mM EDTA as the extractant gave better TP recoveries, but resulted in degradation of the labile Po components (DNA-P and lipid-P). The combination of 0.25 M NaOH and 50 mM EDTA has been widely used for analyzing soils, animal manure, and marine and lake sediments [20], [30], [45], and it was chosen as the appropriate extractant for the Po analysis in our river sediment samples.

## Recommended Procedure

1. Freeze dry the sediment, grind it, and pass it through a 100-mesh sieve.
2. Weigh 3.00±0.01 g of the sediment sample and place it in a 50-mL centrifuge tube.
3. Add 30 mL of 0.25 M NaOH and 50 mM EDTA solution (at a sediment:solution ratio of 1∶10) and shake for 16 h at 25°C.
4. Centrifuge the sample at high speed 9462×g (RCF) at 4°C for 30 min, and retain the supernatant.
5. Analyze TP and Pi in an aliquot of the extract using the molybdenum blue method before and after digesting the extract with potassium persulfate (K_2_S_2_O_8_), after diluting the extract 100-fold to avoid EDTA interfering with the determination. Po is calculated as the difference between the TP and Pi. Freeze dry the remaining extract for ^31^P-NMR analysis.
6. Take 300 mg of the lyophilized extract and re-dissolve it in 0.6 mL D_2_O and 0.1 mL of 10 M NaOH. Ultrasonicate the extract for 30 min and then equilibrate it for 5 min. Add 2% (V:V) of bicarbonate buffered dithionite (0.11 M NaHCO_3_ and 0.11 M Na_2_S_2_O_4_) to the extract to decrease interference in the ^31^P-NMR analysis by paramagnetic ions (Fe^3+^ and Mn^2+^).
7. Centrifuge the supernatant for 15 min at 16873×g (RCF) and transfer it to a 5-mm NMR tube for solution ^31^P-NMR analysis.
